# Lorlatinib Effectiveness and Quality-of-Life in Patients with ALK-Positive NSCLC Who Had Failed Second-Generation ALK Inhibitors: Canadian Real-World Experience

**DOI:** 10.3390/curroncol30070481

**Published:** 2023-07-08

**Authors:** Martin Rupp, Fiorella Fanton-Aita, Stephanie Snow, Paul Wheatley-Price, Barbara Melosky, Rosalyn A. Juergens, Quincy Chu, Normand Blais, Shantanu Banerji, Ryan Ng, Shoghag Khoudigian, Arushi Sharma, Phu Vinh On, Geoffrey Liu

**Affiliations:** 1 Pfizer Canada, 17300 Trans-Canada Hwy, Kirkland, QC H9J 2M5, Canada; 2 Queen Elizabeth II Health Sciences Centre, Dalhousie University, 1276 South Park Street, Halifax, NS B3H 2Y9, Canada; 3 Department of Medicine, University of Ottawa, 75 Laurier Avenue East, Ottawa, ON K1N 6N5, Canada; pwheatleyprice@toh.ca; 4 BC Cancer–Vancouver, 600 West 10th Avenue, Vancouver, BC V5Z 4E6BC, Canada; 5 Juravinski Cancer Centre, McMaster University, Hamilton, ON L8V 5C2, Canada; juergensr@hhsc.ca; 6 Cross Cancer Institute, 11560 University Ave, Edmonton, AB T6G 1Z2, Canada; 7 Centre Hospitalier de l’Université de Montréal, Rue Saint-Denis Street Pavillon R, Montreal, QC H2X 0A9, Canada; 8 CancerCare Manitoba Research Institute, Rady Faculty of Health Sciences, University of Manitoba, Winnipeg, MB R3E 0V9, Canada; 9 IQVIA Solutions Canada, 16720 Trans-Canada Highway, Kirkland, QC H9H 5M3, Canada; 10 Princess Margaret Cancer Centre, Temerty School of Medicine, University of Toronto, Toronto, ON M5G 2M9, Canada

**Keywords:** lorlatinib, non-small cell lung cancer, real-world evidence, quality-of-life, effectiveness, second line

## Abstract

Lorlatinib is the only targeted therapy approved in Canada to treat patients with anaplastic lymphoma kinase (ALK)-positive non-small cell lung cancer (NSCLC) whose tumor has progressed despite treatment with second-generation ALK tyrosine kinase inhibitor (TKI), a patient population with high unmet need and lack of publicly reimbursed targeted treatments in Canada. We prospectively examined the real-world effectiveness and impact of lorlatinib on quality-of-life in 59 lorlatinib-treated patients, characterized as: median age of 62.0 years; 47.5% were female; 32.2% had central nervous system metastases; 50.8% had 2+ prior ALK TKI lines; and alectinib was the most common ALK TKI (72.9%) administered before lorlatinib, including 44.1% who received first-line alectinib. With a median follow-up of 15.3 months (IQR: 6.2–19.2), median time-to-treatment discontinuation of lorlatinib was 15.3 months (95% CI: 7.9–not reached), with 54.2% (95% CI: 40.8–65.9%) of patients without treatment discontinuation at 12 months. At baseline, the mean health utility score (HUS) was 0.744 (SD: 0.200). At 3 months, patients receiving lorlatinib demonstrated a 0.069 (95% CI: 0.020–0.118; *p* = 0.007) average HUS increase over baseline; HUS was maintained at 6 and 12 months. Thus, patients with ALK-positive NSCLC post second-generation ALK TKI remained on lorlatinib for a meaningful duration of time while their quality-of-life was preserved.

## 1. Introduction

Non-small cell lung cancer (NSCLC) accounts for the majority of lung cancer cases in Canada [1], with anaplastic lymphoma kinase (ALK)-positive NSCLC occurring in approximately 1–5% of patients with NSCLC [2,3]. The National Comprehensive Cancer Network Clinical Practice Guidelines in Oncology and the European Society for Medical Oncology Clinical Practice Guidelines for NSCLC recommends a second- or third-generation ALK tyrosine kinase inhibitor (TKI) as the preferred first-line treatment option for these patients [4,5]. Despite the efficacy of second-generation ALK TKIs, patients with ALK-positive NSCLC progressing on these agents often present with a high incidence of CNS metastases, ranging from 40–84% in addition to difficult-to-treat ALK resistant mutations [6,7,8,9,10,11,12,13]. Central nervous system (CNS) metastases are associated with significant morbidity and mortality, reduction in quality-of-life, as well as increased healthcare utilization [14,15,16]. Chemotherapy has demonstrated only modest clinical benefit in patients progressing on second-generation ALK TKIs [9] and even less so for single agent immunotherapy [4,17]. Consequently, there is an unmet need for effective targeted treatment in patients with ALK-positive NSCLC progressing on second-generation ALK TKIs.

Lorlatinib is a potent, brain-penetrant, third-generation ALK TKI that addresses mechanisms of resistance following previous treatment with ALK TKIs [11]. In a global Phase II study, lorlatinib demonstrated promising overall and intracranial activity among pre-treated patients with ALK-positive NSCLC (Table 1) [11]. These results led to regulatory approval of lorlatinib by a number of international drug regulatory agencies, including Health Canada, the Food and Drug Administration (FDA), and the European Medical Agency (EMA) [7]. Additionally, in these pre-treated ALK-positive NSCLC settings, lorlatinib received positive reimbursement recommendations in over 25 countries (Supplementary File S1).

In Canada, based on the promising finding of antitumour activity observed in the Phase II study showing that lorlatinib addresses high unmet need in patients pretreated with second-generation ALK TKIs, lorlatinib received regulatory approval [7] and currently remains the only targeted therapy approved in this setting. However, lorlatinib received a negative Canadian Health Technology Assessment (HTA) recommendation for the treatment of adult patients with pre-treated ALK-positive metastatic NSCLC owing to the uncertainty of the Phase II non-randomized data [18,19]. As a result, lorlatinib is not publicly reimbursed for this patient population in Canada.

Since the global Phase II trial read-out, the clinical benefit of lorlatinib in the post second-generation setting has been confirmed in multiple real-world cohorts with median PFS and duration of treatment (DoT) ranging from 6.2–9.9 months and 4.4–11.8 months, respectively (Table 1) [20,21,22,23,24,25,26,27]. However, chemotherapy and immunotherapy (median PFS in a range of 2–3 months) remain the only publicly reimbursed therapeutic options for Canadian patients progressing on second-generation ALK TKIs [9,17].

**Table 1:** Median Progression-Free Survival (PFS) and Duration of Treatment (DoT) of lorlatinib—data from clinical studies and real-world studies in patients pre-treated with second-generation ALK TKIs.

Study Type	*n*	Median PFS in Months (95% CI)	Median DoT in Months [Range, Unless Otherwise Stated]
Cohort Exp 3B-5, Phase 2 study (NCT01970865)—Multi-country/Global [28]	139	6.6 (5.4–7.4)	10.1 [0.2–43.2]
Cohort 2, Phase 2 study (NCT03909971)—People’s Republic of China [29]	42	5.6 (2.9–9.7)	8.43 [range: 0.7–14.9] *
RWE Study—France [22]	195	9.9 (6.0–12.3)	11.8 [95% CI: 8.5–18.8]
RWE Study—Hong Kong, Singapore, South Korea, Taiwan, Thailand, and the United States [23]	76	9.3 (6.5–not reached)	5.6 [0–24.7]
RWE Study—Taiwan [24]	22	6.2 (4.2–8.3)	Not reported
RWE Study—Korea [25]	10	6.5 (1.0–16.5) ^#^	5.8 [1.3–16.5] ^#^
RWE Study—Germany [26]	37	7.1 (4.9–9.3)	10.4 [95% CI: 6.5–12.8] ^$^
RWE Study—Austria [27]	37	Not reported	4.4 [95% CI: 1.3–7.6]
RWE Study—Japan [20]	51	Not reported	11.1 [95% CI, 4.6–13.8] **
RWE Study—Korea [21]	90	7.5	Not reported

* median treatment exposure; ^#^ data includes 2 ROS1 NSCLC patients (*n* = 12); ^$^ data includes ROS1 NSCLC patients (*n* = 52); ** time to treatment failure endpoint. CI, confidence interval; PFS, median progression-free survival; DoT, duration of treatment; RWE, real-world evidence.

To allow Canadian patients with ALK-positive NSCLC progressing on second-generation ALK TKIs to have access to targeted therapy, a time-limited Canadian patient access program for lorlatinib was operated by Pfizer Canada ULC. Leveraging lorlatinib’s access program infrastructure, we assessed the real-world outcomes of lorlatinib after failure of second-generation ALK TKIs.

## 2. Methods

### 2.1. Study Design and Study Population

This is a prospective cohort study utilizing real-world data. The population consisted of Canadian patients with ALK-positive NSCLC who had failed second-generation ALK TKI, and whose physician sought access to lorlatinib through an access program (August 2020–May 2021). In this program, patients received lorlatinib regardless of whether they participated in the study. Only patients who provided consent for data collection were included in the analysis. Patients who enrolled in the access program but did not receive lorlatinib were all excluded from all analyses. All patients received lorlatinib in accordance with the Health Canada-approved Product Monograph indication in the post-second-generation ALK TKI setting [7]. This protocol was approved by the Advarra Institutional Review Board (A-IRB) and all analyses were pre-specified.

### 2.2. Follow-Up and Data Sources

This project involved primary data collection and secondary use of de-identified patient data. The date of lorlatinib initiation was the index date. Patients were followed for the duration of participation in the access program until they either withdrew consent, discontinued lorlatinib, or died.

Baseline demographic information was collected from the patient and clinical information from the physician. Dates of dispensing of lorlatinib, discontinuation date, and reason for lorlatinib discontinuation were routinely collected and validated by the access program through a drug access navigator, healthcare provider, pharmacist, and/or directly from the patient.

Each consented patient was also contacted for up to four telephone interviews over the course of one year to capture patient’s quality-of-life at: (i) baseline, prior to lorlatinib initiation or right after lorlatinib initiation (up to seven days after); (ii) three months after lorlatinib initiation (up to 6 weeks after three months); (iii) six months after lorlatinib initiation (up to 6 weeks after six months); and (iv) twelve months after lorlatinib initiation (up to 6 weeks after twelve months).

Quality-of-life interviews were only conducted on patients who were still alive and had not discontinued lorlatinib or withdrawn consent. Follow-up continued with data cut-off date of 10 October 2022.

### 2.3. Outcomes and Covariates

The two primary endpoints assessed in this project were time-to-treatment discontinuation (TTD) and changes in patient quality-of-life from baseline. TTD was defined as the time from lorlatinib therapy start date (i.e., index date) until lorlatinib discontinuation (end of lorlatinib therapy) or death. Where patients and physicians did not respond to requests for updates and no further refill requests were made, discontinuation dates were defined if an individual had a gap in lorlatinib therapy of more than 30 days from the end of a lorlatinib prescription (based on pill count) to the next refill date; that date was defined as 30 days from the end of a lorlatinib prescription. As lorlatinib was only made available every 30 days after confirmation by the patient that the drug was still being prescribed, and confirmed by physician every 90 days for continued benefit, these dates were accurate within a 30-day window. A subject was censored if they withdrew consent or remained on lorlatinib as of the data cut-off date (10 October 2022).

The patient health utility score (HUS) is a single measure value that considers the preference of an individual in a particular health state which serves to estimate quality-of-life [30,31]. HUS values were captured over telephone interview using the 5-Level EuroQol 5-Dimension (EQ-5D-5L) questionnaire [32]. The EQ-5D-5L questionnaire consists of the EQ-5D descriptive system (mobility, self-care, usual activities, pain/discomfort, and anxiety/depression) with an accompanying visual analogue scale (EQ-VAS). Each of these dimensions has five levels indicating no problems, slight problems, moderate problems, severe problems, and extreme problems. The EQ-5D-5L questionnaire has one question per dimension with the five levels corresponding to potential responses; the completed responses to the five dimensions questions are then used to determine the HUS. The EQ-5D-5L has been found to have good reliability and sensitivity with the potential to detect 3125 unique health states [33]. The HUS was calculated using the Canadian tariff (i.e., Canadian-specific quality-of-life preference weights), with the HUS ranging from −0.148 for the worst (55555) to 0.949 for the best (11111) EQ-5D-5L states [31].

Demographics were reported at index in terms of sex (female/male) and age (<65 years/65+ years). Clinical characteristics were also reported at index, including CNS metastases at enrolment (yes/no/not evaluated) and Eastern Cooperative Oncology Group (ECOG) Performance Status (0/1/2+). Prior treatment information collected from all patients included number of prior ALK TKI therapies (1/2+) and last ALK TKI received before lorlatinib. To provide more context on the patients enrolled in the patient access program, the protocol was amended during patient recruitment to collect for all subsequently enrolled patients additional treatment and clinical information (i.e., time from diagnosis of metastatic ALK-positive NSCLC to lorlatinib initiation, duration of therapy of the last ALK TKI received, last line of therapy discontinuation reason, total duration of therapy for prior systemic therapies).

### 2.4. Analysis

TTD was estimated with survival analysis using Kaplan–Meier methodology (with 95% confidence intervals [CI]). Patient HUS at baseline was compared to the HUS at 3, 6 and 12 months post-lorlatinib initiation among patients still receiving lorlatinib for 3, 6, and 12 months, respectively. Statistically significant changes in the HUS were calculated using paired t-tests and reported with 95% CI. Based on US-based HUS change for lung cancer, a change in HUS of 0.06 during follow-up compared to baseline indicated a clinically significant change in quality-of-life [34].

Categorical variables were reported as the absolute number of patients falling into each category and as a percentage of all subjects. Continuous variables were reported in terms of median and interquartile range (IQR), minimum and maximum, as well as the proportion missing. All analyses were conducted using SAS v9.4. As the nature of the project was descriptive, all statistical testing was interpreted as being exploratory and adjustments for multiple comparisons were not performed.

### 2.5. Stratified Analysis

The TTD and change in quality-of-life endpoints at 3, 6, and 12 months were calculated and stratified by the: (i) number of lines of ALK TKI therapy prior to lorlatinib initiation (1 vs. 2+); (ii) presence of CNS metastases at enrolment (yes vs. no/not evaluated); (iii) baseline ECOG status (0/1 vs. 2+); and (iv) last ALK TKI received (alectinib vs. other [crizotinib, brigatinib, and ceritinib]). Results were further examined for the subset of patients who had only received first-line alectinib.

## 3. Results

**Figure 1:**
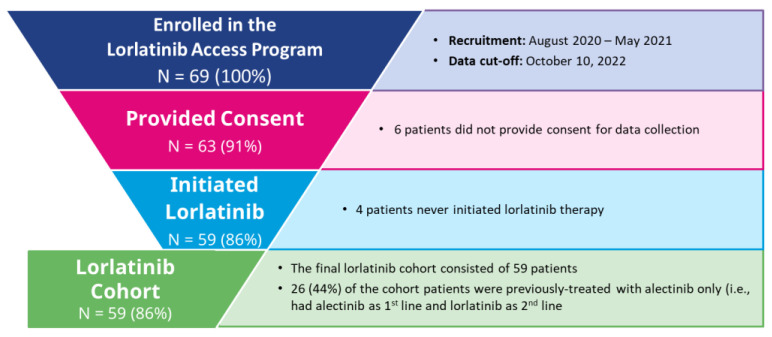
Lorlatinib Cohort Inclusion/Exclusion Criteria.

Of 69 enrolled patients with ALK-positive NSCLC pretreated with second-generation ALK TKIs, 59 patients (85.5%) consented to data collection and initiated lorlatinib treatment—these patients comprised the lorlatinib cohort (Figure 1).

### 3.1. Baseline Characteristics

At baseline, the median age of the cohort was 62.0 (IQR = 55.0–69.0) years; 47.5% were female (Table 2). The patients enrolled were from six Canadian provinces, with most patients enrolled from Ontario (30.5%), British Columbia (27.1%), Alberta (20.3%), and Quebec (15.3%). CNS metastases were present in 32.2% of patients and most of the patients (88.1%) had an ECOG Performance Status (PS) of 0 or 1. The majority of patients (88.1%) initiated lorlatinib at the recommended dose of 100 mg per day, while the remaining patients either initiated treatment at lower doses (50–75 mg per day), or the information on initial lorlatinib dose was missing. The median time from diagnosis of metastatic ALK-positive NSCLC to initiation of lorlatinib therapy was 16.7 (IQR = 11.0–28.0) months. Approximately half (50.8%) of the patients received two or more ALK TKIs prior to lorlatinib. Alectinib was the most common prior ALK TKI (*n* = 43; 72.9%) before lorlatinib initiation, including 44.1% of patients (*n* = 26) who had only received first-line alectinib. Of the 15 patients who received first-line alectinib and for whom information was available, the median time from diagnosis of metastatic ALK-positive NSCLC to lorlatinib initiation was 13.4 months (95% CI: 8.8–16.7), while the median duration of therapy on alectinib was 12.0 months (95% CI: 7.0–15.0). Lastly, the baseline demographics and clinical characteristics of patients, for whom additional treatment and clinical information was collected (*n* = 30), was comparable to that of the patients for whom the additional information was not collected (*n* = 29).

### 3.2. Time-to-Treatment Discontinuation (TTD)

**Figure 2:**
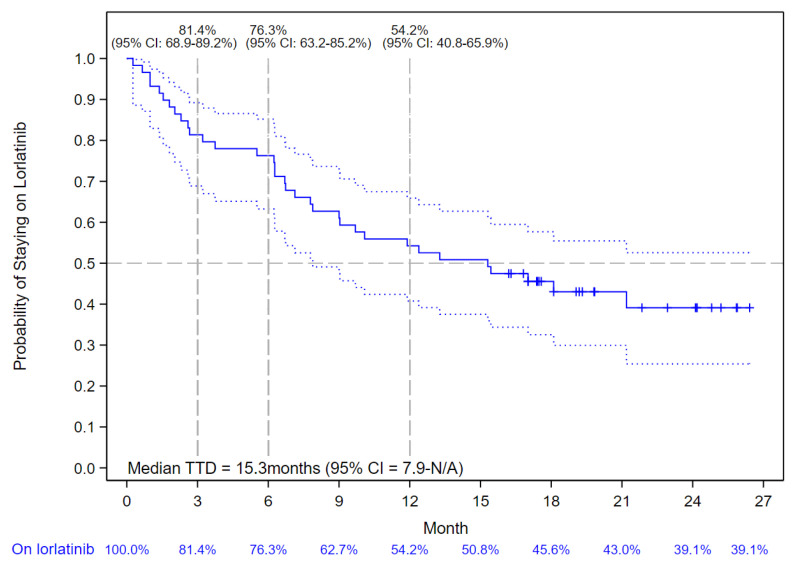
Kaplan–Meier Curve of Lorlatinib Time-to-Treatment Discontinuation (TTD). The solid blue line indicates the Kaplan-Meier Curve values with vertical bar markers indicating censoring; the dotted blue lines indicate the 95% confidence intervals.

Among the 59 patients enrolled in the lorlatinib cohort, the median duration of follow-up was 15.3 months (IQR: 6.2–19.2). The median TTD of lorlatinib treatment was 15.3 months (95% CI: 7.9–not reached) with 81.4% (95% CI: 68.9–89.2%), 76.3% (95% CI: 63.2–85.2%), and 54.2% (95% CI: 40.8–65.9%) of patients without treatment discontinuation or death at 3, 6, and 12 months, respectively (Figure 2).

**Figure 3:**
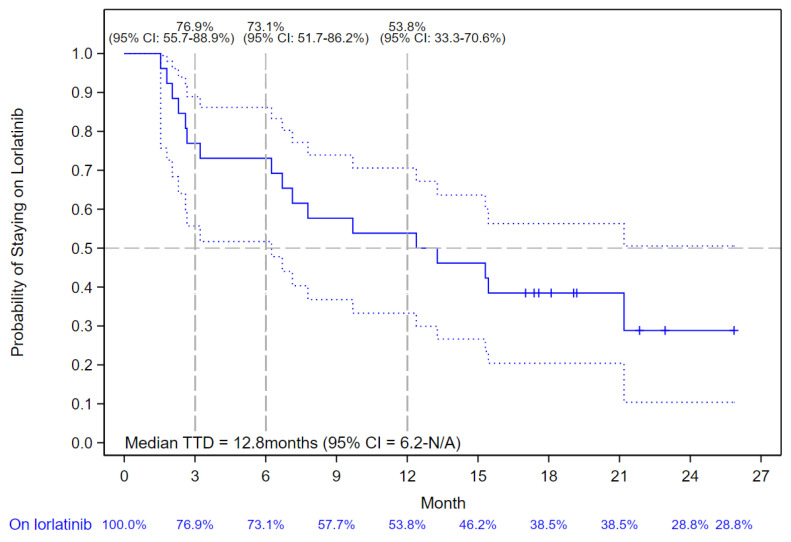
Kaplan–Meier Curve of Lorlatinib Time-to-Treatment Discontinuation (TTD) in patients who Received First-line Alectinib. The solid blue line indicates the Kaplan-Meier Curve values with vertical bar markers indicating censoring; the dotted blue lines indicate the 95% confidence intervals.

In patients with a history of first-line alectinib treatment only (*n* = 26), the median TTD of lorlatinib was 12.8 months (95% CI: 6.2–not reached) (Figure 3).

The median TTD of lorlatinib in patients with one prior ALK TKI was 15.3 months while patients with two or more lines of prior ALK TKIs had a median TTD of 14.5 months. In patients with baseline CNS metastases, the median TTD of lorlatinib had not been reached, while in those without baseline CNS metastases, the median TTD of lorlatinib was 12.1 months. Patients with an ECOG PS of 0 or 1 at the initiation of lorlatinib had a median TTD of lorlatinib of 15.4 months, while patients with an ECOG PS of 2 or higher had a median TTD of 6.3 months. Lastly, in patients whose last ALK TKI was alectinib, the median TTD of lorlatinib was 13.3 months, while in patients who had received another ALK TKI as their last therapy the median TTD of lorlatinib was 18.1 months (Table 3).

In patients who discontinued lorlatinib, reasons for lorlatinib discontinuation were as follows: ten patients (16.9%) died, seven patients (11.9%) experienced disease progression, and fewer than five patients (<8.5%) were categorized into each of the following categories: gap of greater than 30 days between prescription refills, patient decision, side effect, decided to try an alternative therapy, or other reason.

### 3.3. Change in Patients’ Health Utility Score

**Figure 4:**
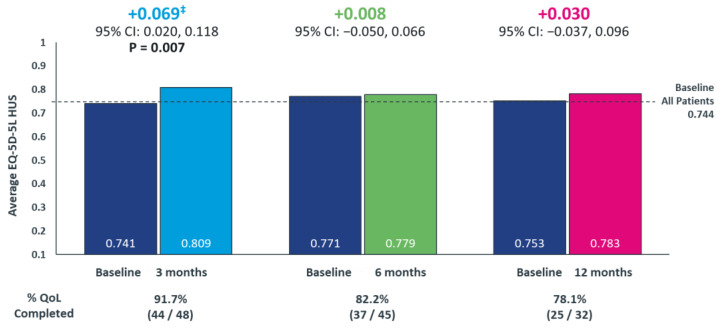
Longitudinal Assessment of the Change in Patients’ Quality-of-Life from Baseline Following Lorlatinib initiation using EQ-5D-5L questionnaire ^‡^ A 0.06 change in HUS is considered a minimally clinically-important difference for US-based utility value for cancer populations [34]. EQ-5D-5L, 5-Level EuroQol 5-Dimension; health utility index score, HUS. *p*-values only shown where statistically significant differences were observed.

At baseline, the mean HUS (*n* = 59) was 0.744 (SD = 0.200) and mean self-rated health score was 65.2 (SD = 23.0). Among patients who received lorlatinib therapy, the HUS questionnaire completion rate at 3, 6, and 12 months was 91.7% (44/48), 82.2% (37/45), and 78.1% (25/32), respectively. Patients demonstrated a statistically significant and clinically meaningful increase in HUS of 0.069 (95% CI, 0.020–0.118) (*p* = 0.007) at 3 months compared to baseline. Patients maintained HUS, with an increase of 0.008 (95% CI, −0.050–0.066) and 0.030 (95% CI, −0.037–0.096) at 6 and 12 months, respectively, compared to baseline (Figure 4).

Across all stratifications, the HUS values were either improved or were maintained compared to baseline (Table 4). Among patients with two or more lines of prior ALK TKIs, a significant increase (*p* = 0.016) in HUS from 0.695 at baseline to 0.795 at 3 months was observed.

Overall, patients with CNS metastases reported a lower HUS at baseline than those without CNS metastases. Among the patients with baseline CNS metastases, compared to baseline, there was an increase in HUS of 0.131, 0.071, and 0.054 at 3, 6, and 12 months, respectively, with the increase at 3 months being statistically significant (*p* = 0.047).

Similarly, patients with ECOG PS of 2 or higher had a lower HUS at baseline than those with ECOG PS 0 or 1. Among the patients with baseline ECOG PS of 2 or higher, compared to baseline, there was a clinically meaningful increase in HUS of 0.267, 0.317, and 0.250 at 3, 6, and 12 months, respectively.

Lastly, patients with either alectinib or other ALK TKIs as their last ALK TKI therapy reported similar HUS at baseline. The baseline HUS among patients who received alectinib as their last ALK TKI was 0.754, which increased by 0.064 at 3 months representing a statistically significant and clinically meaningful increase (*p* = 0.048); at 6 and 12 months, HUS was maintained from baseline.

## 4. Discussion

In this Canadian RWE prospective cohort study, we evaluated lorlatinib effectiveness and impact on quality-of-life in the post-second generation ALK TKI setting, a rare patient population with high unmet need. The cohort was distributed geographically across Canada with baseline characteristics corresponding to typical patient populations in these settings [11,28,35].

The endpoint, TTD, is a proposed pragmatic clinical endpoint for RWE studies due to its strong correlation with PFS [36,37,38]. In our cohort, the median lorlatinib TTD was 15.3 months, which compares favourably to lorlatinib’s clinical trial (mPFS: 5.6–6.6 months; mDoT: 10.1 months) and other real-world observational study outcomes (mPFS: 6.2–9.9 months; mDoT: 4.4–11.8 months) [20,21,22,23,24,25,26,27,28,29], despite baseline demographics of the studies being generally comparable. For example, the median age ranged from 51–61 years; a nearly equal proportion of males and females participated; more than 70% of patients had ECOG PS of 0–1; and the percentage of patients with CNS metastasis ranged from 28%–75% [20,21,22,23,24,25,26,27]. TTD was consistent across all subgroup analyses, including line of therapy, last ALK TKI received, ECOG PS, and presence or absence of CNS metastases at lorlatinib initiation. While the reasons for prescribing lorlatinib therapy were not available, one explanation could be hesitation to use other therapies (e.g., platinum-based chemotherapy) that have modest clinical benefit [9].

We explored lorlatinib TTD in patients who failed first-line alectinib and found a substantially shorter duration of first-line alectinib than in previously reported studies (12.0 vs. 28.1 months) [39], suggesting enrichment of poor prognostic patients. Despite this, lorlatinib demonstrated clinically meaningful outcomes as the median TTD of lorlatinib in this post-first-line alectinib population was comparable to the overall cohort (12.8 vs. 15.3 months). Overall, these findings further support lorlatinib effectiveness in all subgroups of patients who had failed second-generation ALK TKI, a patient population that historically has derived only very limited benefit from chemotherapy [9].

A quality-of-life surrogate, the EQ-5D-5L questionnaire, has been found to have good reliability and sensitivity [33]. We observed statistically and clinically meaningful improvements from baseline in quality-of-life at 3 months and quality-of-life on lorlatinib continued to be maintained at 6 and 12 months, results consistent with other studies [35,40,41]. Lorlatinib improved quality-of-life in those patients with worse initial quality-of-life as measured by lower baseline HUS, i.e., patients with CNS baseline metastases and poor ECOG PS. The improved quality-of-life results in patients with baseline CNS metastases complement findings observed in the CROWN first-line trial [40]. Having good (0 or 1) or poor (≥2) ECOG PS at baseline led to similar TTD outcomes (*p* = 0.381). Further, patients with poor (≥2) ECOG PS at baseline had a clinically meaningful improvement in quality-of-life on lorlatinib at 3, 6, and 12 months vs. baseline, which confirmed data reported from another RWD study [42].

Although randomized controlled clinical trials are still considered the gold standard in providing evidence related to efficacy and safety of drugs, there are certain scenarios where RWE may provide novel, supplemental information about clinical effectiveness, safety, cost effectiveness, and/or budget impact not addressed by the clinical data [43,44]. In recent times, regulatory bodies, HTA agencies, and other stakeholders have recognized the importance of RWE in addressing the evidence gaps for decision-making for drugs, with new frameworks being developed [45,46]. We utilized a novel RWE strategy of embedding the voluntary collection of patient data by leveraging a patient access program infrastructure. Innovative, forward-thinking strategies are needed to incorporate the critical appraisal and consideration of RWE, such as this study, into processes that lead to funding decisions. Process improvements would help reduce the disparity and inequity felt by cancer patients who do not have the means to pay (e.g., private insurance) for costly drug therapies when RWE demonstrates its clinical benefits [47]. The high participation rate of patients enrolled in the patient access program who consented to the project (>85%) also demonstrates the feasibility of RWE studies, the willingness of patients in supporting RWE and their contributions to the research process should not be overlooked. To communicate scientific findings in this manuscript to a broader audience, we have developed a supplementary lay abstract (Supplementary Files S2 and S3) and plain language summary (Supplement Files S4 and S5) in English and French that includes contributions and review by two Canadian patients living with ALK-positive NSCLC.

Leveraging patient access program infrastructure to collect RWE is a major strength of this project. Instead of defining TTD based on gaps in prescription refills, discontinuation date, and reason for discontinuation were readily captured and validated through multiple sources (it is helpful that lorlatinib supply comes from the access program), which contribute to accurate estimation of TTD. Embedding collection of a validated quality-of-life single-measure surrogate, the EQ-5D, is also an advancement of RWE, thus allowing the capture of quality-of-life with high completion rates of >78%.

The presented results have several limitations, including a lack of evaluation of lorlatinib safety; however, there were fewer than five patients (<8.5%) who discontinued lorlatinib due to adverse events. Additionally, because amendments to capture additional data were made in the middle of the project (a sign of the learning curve in designing novel prospective RWE studies), the duration of treatment on prior ALK TKIs and time from metastatic disease to lorlatinib initiation was not available in approximately half of patients. Thirdly, a small sample size is due to the rarity of the indication, which deems interpretation of the stratified results as exploratory. Lastly, receiving lorlatinib through the patient access program itself may have potentially biased the cohort. However, the majority of Canadian ALK-positive patients could only access lorlatinib through this access program (only a few private insurers cover lorlatinib) and there was a high participation rate among patients who entered the access program.

## 5. Conclusions

Our analysis demonstrated the feasibility of RWE generation leveraging the lorlatinib access program infrastructure by examining clinically meaningful outcomes in Canadian patients. Results were consistent with other clinical trials and RWE studies conducted in the post-second generation ALK TKIs setting. Collaborations between industry and stakeholders (Health Canada, the Canadian Agency for Drugs and Technologies in Health [CADTH], Institut national d’excellence en santé et en services sociaux [INESSS], the pan-Canadian Pharmaceutical Alliance [pCPA], provincial payers, academic oncologists) are needed to ensure timely patient access to therapies for high unmet need patient populations.

## Figures and Tables

**Table 2:** Patient Demographic and Clinical Characteristics and Treatment History at Baseline.

Baseline Characteristic	Lorlatinib Cohort *n* = 59
**Age at Lorlatinib Initiation (Years)**	
Median (IQR)	62 (55–69)
Minimum–Maximum	35–89
**Age group, *n* (%)**	
<65 years	36 (61.0%)
65+ years	23 (39.0%)
**Sex, *n* (%)**	
Female	28 (47.5%)
Male	31 (52.5%)
**CNS Metastases, *n* (%)**	
Yes	19 (32.2%)
No or Not evaluated ^	40 (67.8%)
**ECOG Performance Status, *n* (%)**	
0 or 1	52 (88.1%)
2+	7 (11.9%)
**Number of Prior ALK TKI Therapies, *n* (%)**	
1	29 (49.2%)
2+	30 (50.8%)
**Last ALK TKI Received Before Lorlatinib, *n* (%)**	
Alectinib	43 (72.9%)
Brigatinib	10 (16.9%)
Others (ceritinib or crizotinib) ^#^	6 (10.2%)
**Lorlatinib Initial dose, *n* (%) ^†^**	
100 mg	52 (88.1%)
50 mg or 75 mg	7 (11.9%)
**Duration of Therapy of the Last ALK TKI Received (Months)**	
Median (IQR)	12 (9–16)
Minimum–Maximum	3–44
Not available, *n* (%)	32 (54.2%) *
**Time from Diagnosis of metastatic ALK-positive NSCLC to lorlatinib initiation (Months)**	
Median (IQR)	16.7 (11.0–28.0)
Minimum–Maximum	5.0–91.0
Not available, *n* (%)	36 (61.0%) *
**Last Line of Therapy Discontinuation Reason, *n* (%)**	
Disease Progression	23 (39.0%)
Tolerability/Adverse Events/Other	6 (10.2%)
Not available	30 (50.8%) *
**Total Duration of Therapy for Prior Systemic Therapies (including ALK TKIs) (Months)**	
Median (IQR)	15.5 (11.5–26.5)
Minimum–Maximum	3–54
Not available, *n* (%)	31 (52.5%) *

^ There were under 5 patients who were not evaluated for CNS lesions. ^#^ Patients who have received crizotinib have also received prior second-generation ALK TKI (ceritinib, alectinib, or brigatinib). ^†^ Information missing for <5 patients. * This information was not available for all patients because it was only collected part way through the study in prospectively recruited patients after a protocol amendment. ALK, anaplastic lymphoma kinase; CNS, central nervous system; ECOG, Eastern Cooperative Oncology Group; IQR, interquartile range; PAP, patient access program; SD, standard deviation; TKIs, tyrosine kinase inhibitors.

**Table 3:** Median Time-to-Treatment Discontinuation (TTD) of Lorlatinib by Stratification Groups.

Stratification	Median TTD (95% CI)	Log-Rank Test
**Number of Prior Lines of ALK TKIs**		
1 prior line (*n* = 29)	15.3 months (6.7–not reached)	*p* = 0.964
2+ prior lines (*n* = 30)	14.5 months (6.7–not reached)	
**Presence of CNS Metastases**		
Yes (*n* = 19)	Not reached	*p* = 0.190
No or not evaluated ^^^ (*n* = 40)	12.1 months (6.7–21.2)	
**ECOG PS**		
0/1 (*n* = 52)	15.4 months (9.0–not reached)	*p* = 0.381
2+ (*n* = 7)	6.3 months (0.7–not reached)	
**Last ALK TKI Received Before Lorlatinib**		
Alectinib (*n* = 43)	13.3 months (7.1–not reached)	*p* = 0.564
Other ALK TKI ^†^ (*n* = 16)	18.1 months (6.3–not reached)	

^ There were under 5 patients who were not evaluated for CNS metastases. ^†^ Other ALK TKI consisted of brigatinib, crizotinib, and ceritinib. ALK, anaplastic lymphoma kinase; CNS, central nervous system; ECOG PS, Eastern Cooperative Oncology Group Performance Status; TKIs, tyrosine kinase inhibitors; TTD, time-to-treatment discontinuation.

**Table 4:** Longitudinal Assessment of the Change in Health Utility Index Score (HUS) from Baseline Following Lorlatinib initiation Based on Stratifications at 3, 6, and 12 months.

Stratification	Study Cohort with at Least 3 Months of Follow-Up			Study Cohort with at Least 6 Months of Follow-Up			Study Cohort with at Least 12 Months of Follow-Up		
	Mean HUS at Baseline	Mean HUS at 3 Months	Mean Change from Baseline (95% CI)	Mean HUS at Baseline	Mean HUS at 6 Months	Mean Change from Baseline (95% CI)	Mean HUS at Baseline	Mean HUS at 12 Months	Mean Change from Baseline (95% CI)
**Number of Prior Lines of ALK TKIs (% of patients who completed HUS questionnaire)**									
1 prior line	0.795 (87%)	0.827 (87%)	+0.032 (−0.023–0.086)	0.813 (81.8%)	0.812 (81.8%)	−0.001 (−0.051–0.049)	0.828 (50%)	0.857 (50%)	+0.028 (−0.058–0.115)
2+ prior lines	0.695 (96%)	0.795 (96%)	+0.100 ^‡^ (0.020–0.180) (*p* = 0.016)	0.730 (82.6%)	0.747 (82.6%)	+0.016 (−0.092–0.124)	0.694 (90%)	0.725 (90%)	+0.030 (−0.078–0.139)
**Presence of CNS Metastases (% of patients who completed HUS questionnaire)**									
No ^	0.778 (90.6%)	0.815 (90.6%)	+0.037 (−0.001–0.074)	0.812 (83.3%)	0.789 (83.3%)	−0.023 (−0.091–0.046)	0.826 (70%)	0.836 (70%)	+0.010 (−0.067–0.087)
Yes	0.667 (93.8%)	0.799 (93.8%)	+0.131 ^‡^ (0.002–0.261) (*p* = 0.047)	0.685 (80%)	0.757 (80%)	+0.071 ^‡^ (−0.041–0.184)	0.661 (91.7%)	0.715 (91.7%)	+0.054 (−0.078–0.185)
**ECOG Performance Status (% of patients who completed HUS questionnaire)**									
0/1	0.796 (>85%)	0.839 (>85%)	+0.043 (not reported) (*p* = 0.050)	0.816 (60–90%)	0.796 (60–90%)	−0.019 (not reported)	0.820 (60–90%)	0.820 (60–90%)	−0.001 (not reported)
2+	0.309 (100%)	0.576 (100%)	+0.267 ^‡^ (not reported)	0.262 (60–80%)	0.579 (60–80%)	+0.317 ^‡,#^ (not reported)	0.262 (100%)	0.512 (100%)	+0.250 ^‡,#^ (not reported)
**Last ALK TKI Received Before Lorlatinib (% of patients who completed HUS questionnaire)**									
Alectinib	0.754 (88.2%)	0.818 (88.2%)	+0.064 ^‡^ (0.000–0.128) (*p* = 0.048)	0.772 (81.3%)	0.798 (81.3%)	+0.027 (−0.028–0.081)	0.761 (73.9%)	0.807 (73.9%)	+0.046 (−0.033–0.125)
Other ALK TKI	0.712 (100%)	0.791 (100%)	+0.078 ^‡^ (−0.006–0.163)	0.769 (84.6%)	0.732 (84.6%)	−0.037 (−0.200–0.127)	0.737 (88.9%)	0.731 (88.9%)	−0.006 (−0.158–0.147)

The mean baseline HUS for the entire PAP cohort (*n* = 59) was 0.744. ^ There were under 5 patients who were not evaluated for CNS metastases. ^‡^ A 0.06 change in HUS is considered a minimally clinically-important difference for US-based HUS for cancer populations. # *p* value not reported. ALK, anaplastic lymphoma kinase; CNS, central nervous system; ECOG, Eastern Cooperative Oncology Group; HUS, health utility index score; TKIs, tyrosine kinase inhibitors. *p*-values only shown where statistically significant differences were observed.

## Data Availability

Pfizer will provide access to individual de-identified participant data and related study documents (e.g., protocol, Statistical Analysis Plan (SAP), Clinical Study Report (CSR)) upon request from qualified researchers, and subject to certain criteria, conditions, and exceptions. Further details on Pfizer’s data sharing criteria and process for requesting access can be found at: https://www.pfizer.com/science/clinical-trials/trial-data-and-results/data-requests (accessed on 14 October 2022).

## Supplementary Materials

The following supporting information can be downloaded at: https://www.mdpi.com/article/10.3390/curroncol30070481/s1. Supplementary File S1: List of countries in which lorlatinib received positive reimbursement recommendation as a second-line or subsequent lines of therapy in ALK-positive metastatic NSCLC; Supplementary File S2: Lay abstract in English; Supplementary File S3: Lay abstract in French; Supplementary File S4: Plain Language Summary—infographic in English; Supplementary File S5: Plain Language Summary—infographic in French.
