# Supplementary material for: Lorlatinib Effectiveness and Quality-of-Life in Patients with ALK-Positive NSCLC Who Had Failed Second-Generation ALK Inhibitors: Canadian Real-World Experience
Source: Curr Oncol. 2023 Jul 8;30(7):6559–74. doi: 10.3390/curroncol30070481 (PMC10377946; doi:10.3390/curroncol30070481)
Supplement: Supplementary file 1 [file curroncol-30-00481-s001.zip › Supplementary File S1_Countries_revised.pdf]

List of countries in which lorlatinib received positive reimbursement recommendation as a second-line or subsequent lines of therapy in ALK-positive metastatic NSCLC

	Country	Reference
1	UK	https://www.nice.org.uk/guidance/indevelopment/gid-ta10317
2	Germany	https://www.g-ba.de/bewertungsverfahren/nutzenbewertung/464/
3	France	https://www.has-sante.fr/jcms/p_3146314/fr/lorviqua
4	Italy	Gazzetta Ufficiale
5	Spain	https://www.aemps.gob.es/medicamentosUsoHumano/informesPublicos/docs/2021/IPT_6-2021-Lorviqua.pdf?x69995
6	Poland	https://bipold.aotm.gov.pl/assets/files/zlecenia_mz/2020/085/REK/24%2008%202020%20rekomendacja%20nr%2044%20Lorviqua.pdf
7	Australia	http://www.pbs.gov.au/industry/listing/elements/pbac-meetings/pbac-outcomes/2019-11/positive-recommendations-11-2019.docx.pdf
8	Belgium	https://webappsa.riziv-inami.fgov.be/SSPWebApplicationPublic/fr/Public/ProductSearch
9	Luxembourg	https://cns.public.lu/en/legislations/textes-coordonnes/liste-positive.html
10	Netherlands	https://www.horizonscangeneesmiddelen.nl/geneesmiddelen/lorlatinib-oncologie-en-hematologie-longkanker/versie1
11	Austria	Pfizer Data on File
12	Sweden	https://www.tlv.se/beslut/beslut-lakemedel/begransad-subvention/arkiv/2019-09-30-lorviqua-ingar-i-hogkostnadsskyddet-med-begransning.html
13	Denmark	https://medicinraadet.dk/anbefalinger-og-vejledninger/laegemidler-og-indikationsudvidelser/i-l/lorlatinib-lorviqua-ikke-smacellet-lungekraeft-alk-positiv
14	Norway	https://nyemetoder.no/Documents/Rapporter/Lorlatinib%20(Lorviqua)_ID2018_092-%20hurtig%20metodevurdering.pdf
15	Finland	https://www.kela.fi/laake1527
16	Ireland	http://www.ncpe.ie/drugs/lorlatinib-lorviqua/
17	Greece	Pfizer Data on File
18	Czech Republic	Pfizer Data on File
19	Slovakia	Pfizer Data on File
20	Portugal	https://extranet.infarmed.pt/INFOMED-fo/detalhes-medicamento.xhtml
21	South Korea	https://www.hira.or.kr/bbsDummy.do?pgmid=HIRAA030014040000&brdScnBltNo=4&brdBltNo=46960&pageIndex=1&pageIndex2=1#none
22	Hungary*	Pfizer Data on File
23	Bulgaria	https://www.ncpr.bg/images/rezume_na_dokladi/2022/26.07.2022/Resume-_LORVIQUA_new.pdf
24	Slovenia	Pfizer Data on File
25	Croatia	Pfizer Data on File
26	Singapore	http://www.ace-hta.gov.sg/our-process-and-methods.html#health-techNology
27	Taiwan	https://www.nhi.gov.tw/Content_List.aspx?n=CD8AE4ECB980299E&topn=5FE8C9FEAE863B46

*Access through the Named Patient Program (NPP). NPP in 2L is bridging solution till national reimbursement is granted to make innovative medicines available in case of declared medical unmet need.
