# Supplementary material for: Lorlatinib Effectiveness and Quality-of-Life in Patients with ALK-Positive NSCLC Who Had Failed Second-Generation ALK Inhibitors: Canadian Real-World Experience
Source: Curr Oncol. 2023 Jul 8;30(7):6559–74. doi: 10.3390/curroncol30070481 (PMC10377946; doi:10.3390/curroncol30070481)
Supplement: Supplementary file 1 [file curroncol-30-00481-s001.zip › Supplementary File S2_Lay abstract_English.pdf]

Lorlatinib effectiveness and quality-of-life in patients with ALK-positive NSCLC who had failed second-generation ALK inhibitors: Canadian real-world experience

Lay Abstract

What was the study about?

ALK-positive (ALK+) non-small cell lung cancer (NSCLC) is a rare type of lung cancer caused by changes (rearrangements) to the ALK gene, a gene that helps cells grow normally. These mutations cause cells to grow too much and too fast leading to lung cancer. ALK+ NSCLC may also spread to other parts of the body called metastasis. ALK+ NSCLC is treated with a type of cancer medication called ALK tyrosine kinase inhibitors (TKI) such as crizotinib, ceritinib, alectinib and brigatinib. In some patients, the cancer gets worse (progression) even after taking these ALK TKIs. Lorlatinib, a newer ALK TKI, can help fight ALK+ NSCLC. In Canada, people must pay for lorlatinib out-of-pocket or through private insurance as lorlatinib is not covered by Canadian provincial public insurance.

What did we do?

Patients in Canada with ALK+ NSCLC could access lorlatinib through a Canadian Patient Access Program opened by Pfizer. While lorlatinib can be given as the first treatment to patients with ALK+ NSCLC, only patients whose disease progressed after taking crizotinib, ceritinib, alectinib or brigatinib could join this program. The doctors applied to the program for their patients. Once enrolled, patients could join the project with their consent. This project wanted to understand if lorlatinib provides benefit to patients in routine clinical settings across Canada by looking at the amount of time for which patients took lorlatinib, and the quality-of-life of patients while taking lorlatinib.

What did we find?

59 patients participated in this project from 2020 to 2022, including a similar number of men and women. In one-third of patients, their cancer had already spread to the brain before starting lorlatinib. Half the patients took lorlatinib for over 15 months and over 40% of patients were still taking lorlatinib at the end of the study. Patients reported their quality-of-life improved after 3 months of treatment. The quality-of-life of these patients was stable after 6 and 12 months of lorlatinib treatment. Based on the results, most patients can take lorlatinib for a meaningful period of time without negatively affecting their quality-of-life.
