# Supplementary material for: Lorlatinib Effectiveness and Quality-of-Life in Patients with ALK-Positive NSCLC Who Had Failed Second-Generation ALK Inhibitors: Canadian Real-World Experience
Source: Curr Oncol. 2023 Jul 8;30(7):6559–74. doi: 10.3390/curroncol30070481 (PMC10377946; doi:10.3390/curroncol30070481)
Supplement: Supplementary file 1 [file curroncol-30-00481-s001.zip › Supplementary File S3_Lay abstract_French.pdf]

L’efficacité du lorlatinib et les effets sur la qualité de vie chez des patients atteints d’un CPNPC ALK-positif ayant progressé suite à la prise des inhibiteurs de l’ALK de deuxième génération : expérience en milieu réel au Canada

mé non scientifique

Sur quoi portait l’étude?

Le cancer du poumon non à petites cellules (CPNPC) ALK-positif est un type de cancer du poumon rare causé par des modifications (réarrangements) du gène *ALK*, un gène qui permet la croissance normale des cellules. Ces mutations provoquent une croissance excessive et plus rapide des cellules; c’est ce qui cause le cancer du poumon. Le CPNPC ALK-positif peut aussi se propager à d’autres parties du corps : c’est ce qu’on appelle les métastases. On traite le CPNPC ALK-positif avec un type de médicaments anticancéreux appelés inhibiteurs de tyrosine kinase (ITK) ciblant l’ALK, comme le crizotinib, le céritinib, l’alectinib et le brigatinib. Chez certains patients, le cancer s’aggrave (progression) malgré la prise d’un de ces ITK de l’ALK. Le lorlatinib est un ITK contre l’ALK de troisième génération qui peut aider à combattre le CPNPC ALK-positif. Comme les régimes d’assurance provinciaux du Canada ne remboursent pas ce médicament, les patients canadiens doivent payer le lorlatinib de leur poche ou se le faire rembourser par une assurance privée.

Qu’avons-nous fait?

Au Canada, les patients atteints d’un CPNPC ALK-positif pouvaient avoir accès au lorlatinib par l’entremise d’un programme canadien d’aide aux patients créé par Pfizer lorsque leur médecin à déterminer le lorlatinib comme choix de traitement. Alors que le lorlatinib peut être administré comme premier traitement aux patients atteints d’un CPNPC ALK-positif, seuls les patients dont la maladie avait progressé après un traitement par le crizotinib, le céritinib, l’alectinib ou le brigatinib pouvaient participer à ce programme. Une fois admis, les patients pouvaient se joindre au projet après consentement. Dans ce projet, nous voulions savoir si le lorlatinib procurait des bienfaits aux patients traités en pratique clinique courante dans l’ensemble du Canada, en observant la durée de leur traitement par le lorlatinib ainsi que leur qualité de vie pendant ce traitement.

Qu’avons-nous découvert?

Cinquante-neuf (59) patients, dont à peu près autant d’hommes que de femmes, ont participé à ce projet entre 2020 et 2022. Chez un tiers des patients, le cancer s’était déjà propagé au cerveau avant de commencer le lorlatinib. La moitié des patients ont pris le lorlatinib pendant plus de 15 mois, et plus de 40% en prenaient encore à la fin de l’étude. Les patients ont signalé qu’après 3 mois de traitement, leur qualité de vie s’était améliorée. La qualité de vie de ces patients est demeurée stable à 6 mois et à 12 mois de traitement par le lorlatinib. Ces résultats indiquent que la plupart des patients peuvent prendre le lorlatinib pendant une période prolongée sans que leur qualité de vie en soit réduite.
