# Supplementary material for: Lorlatinib Effectiveness and Quality-of-Life in Patients with ALK-Positive NSCLC Who Had Failed Second-Generation ALK Inhibitors: Canadian Real-World Experience
Source: Curr Oncol. 2023 Jul 8;30(7):6559–74. doi: 10.3390/curroncol30070481 (PMC10377946; doi:10.3390/curroncol30070481)
Supplement: Supplementary file 1 [file curroncol-30-00481-s001.zip › Supplementary File S4_Infographic_English.pdf]

Lorlatinib effectiveness and quality-of-life in patients with ALK-positive NSCLC who had failed second-generation ALK inhibitors: Canadian real-world experience

In Canada, patients with metastatic ALK-positive (ALK+) NSCLC whose cancer got worse (progressed) despite taking a second-generation ALK TKI can take:

Lorlatinib

- Taken by **mouth**
- **Only targeted therapy** approved in Canada for the above-mentioned settings
- **Not covered in Canada by provincial public insurance** so people must pay for lorlatinib out-of-pocket or with private insurance
- **Lorlatinib** is publicly-insured in **over 25 countries**

Chemotherapy

- **Infusion through veins**
- Data suggests **chemotherapy** **alone** **works for about 3 months** before cancer progresses in patients with ALK+ NSCLC
- **Covered** by Canadian provincial public insurance

Immunotherapy

- **Infusion through veins**
- Data suggests **immunotherapy** **alone** **does not lead to cancer shrinkage** in patients with ALK+ NSCLC
- **Covered** by Canadian provincial public insurance

What did we do?

Who was included?

Patients in Canada with metastatic ALK+ NSCLC whose cancer progressed after taking
- Crizotinib and at least one other ALK TKI, or
- Ceritinib or alectinib

How were the patients enrolled?

Rx Lorlatinib

- Physicians prescribed lorlatinib and **enrolled patients into Patient Access Program**
- After enrollment, **patients could choose to join** in this project with their consent

What was the purpose of this project?

To find out if **lorlatinib provides benefit** to patients with ALK+ NSCLC that progressed after taking a second-generation ALK TKI in routine clinical settings across Canada by looking at:
- **The amount of time for which** patients took **lorlatinib**
- The **quality-of-life** of patients while taking lorlatinib

What type of patients participated?

59 patients participated from 2020–2022. When they joined:
- Half of the patients were 62 years or younger
- Similar number of men and women participated
- One-third of patients already had their cancer spread to the brain
- Half of the patients already received two or more ALK TKIs

What did we find?

Percentage of patients who continued taking lorlatinib at:

3 months	6 months	12 months
**81%**	**76%**	**54%**

- Half of the patients took lorlatinib for over 15 months and over 40% of patients were still taking lorlatinib at the end of the study (average follow-up 15.3 months)

Quality-of-life of patients while taking lorlatinib

Patient quality-of-life **improved** at 3 months

Patient quality-of-life **was stable** at 6 and 12 months

Quality-of-life (Worst – Best)	
Before lorlatinib	0.74
3 months after lorlatinib	0.81
Before lorlatinib	0.77
6 months after lorlatinib	0.80
Before lorlatinib	0.75
12 months after lorlatinib	0.78

Higher scores indicate better health (max: 0.949) and lower scores indicate worse health (min: -0.148)

Take-home message

Based on the results, most patients with ALK+ NSCLC whose cancer progressed on second-generation ALK TKI can take **lorlatinib for a meaningful period of time without negatively affecting their quality-of-life**

More project details

- This is the first project of its kind in Canada to examine how lorlatinib is used in routine clinical practice
- A small number of patients participated in the project because metastatic ALK+ NSCLC is a rare cancer
- Quality-of-life was measured using a health utility score and was collected via patient survey

Want to learn more or get more details?

This manuscript is a plain language summary of the original article:

Rupp M, Fanton-Aita F, Snow S, Wheatley-Price P, Melosky B, Juergens RA, Chu Q, Blais N, Banerji S, Ng R, Khoudigian S, Sharma A, On PV, Liu G (2023) Lorlatinib effectiveness and quality-of-life in patients with ALK-positive NSCLC who had failed second-generation ALK inhibitors: Canadian real-world experience. Current Oncology

*Link: **https://www.mdpi.com/1718-7729/30/7/481***

Key terms

- **ALK (anaplastic lymphoma kinase):** A gene that helps in controlling cell growth.
- **ALK-positive (ALK+) non-small cell lung cancer (NSCLC):** A type of lung cancer caused by changes to the ALK gene (rearrangements) resulting in cells to grow too much and too fast leading to lung cancer. The cancer may spread to other parts of the body (metastasis) like the brain.
- **Targeted therapy**: A type of cancer treatment that targets proteins that control how cancer cells grow, divide, and spread.
- **ALK inhibitors (ALK TKIs):** Cancer treatment that block the action of an enzyme called ALK tyrosine kinase, which may lead to slow or stop cancer growth and shrink the cancer. These medicines are used to treat ALK+ NSCLC.
  - Crizotinib is the oldest ALK TKI (first-generation)
  - Alectinib, brigatinib and ceritinib are second-generation ALK TKIs
  - Lorlatinib is a newer ALK TKI (third-generation)

Disclosure: This study was sponsored by Pfizer Canada ULC. Lorlatinib is approved to treat the condition under study that is discussed in this summary. This summary reports the results of a single study. The results of this study may differ from those of other studies. Health professionals should make treatment decisions based on all available evidence not on the results of a single study. Writing support for this summary was provided by Ryan Ng, Phani Tejasvi Dantu, Shoghag Khoudigian and Arushi Sharma at IQVIA Solutions Canada Inc. and was funded by Pfizer Canada ULC.
