# Supplementary material for: Lorlatinib Effectiveness and Quality-of-Life in Patients with ALK-Positive NSCLC Who Had Failed Second-Generation ALK Inhibitors: Canadian Real-World Experience
Source: Curr Oncol. 2023 Jul 8;30(7):6559–74. doi: 10.3390/curroncol30070481 (PMC10377946; doi:10.3390/curroncol30070481)
Supplement: Supplementary file 1 [file curroncol-30-00481-s001.zip › Supplementary File S5_Infographic_French.pdf]

L’efficacité du lorlatinib et les effets sur la qualité de vie chez des patients atteints d’un CPNPC ALK-positif ayant progressé suite à la prise des inhibiteurs de l’ALK de deuxième génération : expérience en milieu réel au Canada

Au Canada, les patients atteints d’un CPNPC ALK-positif métastatique dont la maladie s’est aggravée (a progressé) malgré l’administration d’un inhibiteur de tyrosine kinase (ITK) de l’ALK de deuxième génération peuvent recevoir un des traitements suivants :

Lorlatinib

- Administration **orale** (par la bouche)
- **Seul traitement** ciblé approuvé au Canada dans le contexte décrit ci-dessus
- **Non remboursé par les régimes d’assurance provinciaux du Canada**; les patients doivent donc le payer de leur poche ou se le faire rembourser par une assurance privée
- **Le lorlatinib** est remboursé par les régimes d’assurance publics de **plus de 25 pays**

Chimiothérapie

- **Administration par perfusion dans une veine**
- Les données indiquent que, chez les patients atteints d’un CPNPC ALK-positif, **la** chimiothérapie **seule serait efficace pendant environ 3 mois** avant que le cancer ne progresse
- **Remboursée** par les régimes d’assurance publics provinciaux du Canada

Immunothérapie

- **Administration par perfusion dans une veine**
- Les données indiquent que, chez les patients atteints d’un CPNPC ALK-positif, **l’immunothérapie seule n’induirait pas une réduction du volume des tumeurs**
- **Remboursée** par les régimes d’assurance publics provinciaux du Canada

Qu’avons-nous fait?

Qui était admis?

Des patients canadiens atteints d’un CPNPC ALK-positif métastatique dont la maladie avait progressé malgré l’administration de :
- crizotinib et au moins un autre ITK ciblant l’ALK; ou
- céritinib ou alectinib.

Comment les patients étaient-ils recrutés?

- Les médecins inscrivaient les patients à qui **ils avaient prescrit le lorlatinib au programme d’aide aux patients**
- Après leur inscription, les **patients pouvaient décider de se joindre** au projet en donnant leur consentement.

Quel était l’objectif de ce projet?

L’objectif était de déterminer si, dans le contexte de la pratique clinique courante dans l’ensemble du Canada, **le lorlatinib procurait des bienfaits** aux patients atteints d’un CPNPC ALK-positif ayant progressé après l’administration d’un ITK de l’ALK de deuxième génération, en observant :
- la **durée des traitements** avec le lorlatinib;
- la **qualité de vie** des patients pendant leur traitement avec le lorlatinib

Quel type de patients ont participé au projet?

Cinquante-neuf (59) patients ont participé au projet entre 2020 et 2022. Au moment de l’admission :
- la moitié des patients étaient âgés de 62 ans ou moins;
- le nombre d’hommes et de femmes était similaire;
- chez un tiers des patients, le cancer s’était déjà propagé au cerveau;
- la moitié des patients avaient déjà reçu au moins deux ITK de l’ALK.

Qu’avons-nous découvert?

Pourcentage de patients qui continuaient à prendre le lorlatinib après :

3 mois	6 mois	12 mois
81%	76%	54%

- La moitié des patients ont pris le lorlatinib pendant plus de 15 mois, et plus de 40 % prenaient encore du lorlatinib à la fin de l’étude (durée moyenne du suivi : 15,3 mois)

Qualité de vie des patients pendant le traitement avec le lorlatinib

La qualité de vie des patients **s’était améliorée** après 3 mois

La qualité de vie des patients **était stable** à 6 mois et à 12 mois

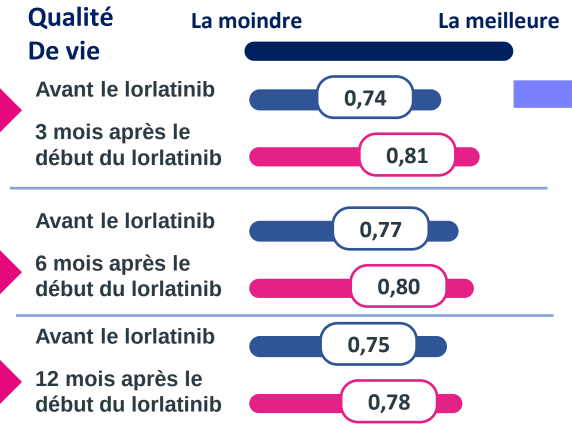

Les scores les plus élevés indiquent un meilleur état de santé (max. : 0,949) et les scores les plus bas indiquent un moins bon état de santé (min. : -0,148)

Message à retenir

Les résultats indiquent que la plupart des patients atteints d’un CPNPC ALK-positif ayant progressé pendant l’administration d’un ITK de l’ALK de deuxième génération peuvent **prendre le lorlatinib pendant une période prolongée sans que leur qualité de vie en soit réduite**

Plus de détails sur le projet

- Il s’agit du premier projet de ce genre au Canada à étudier comment le lorlatinib est utilisé dans la pratique courante
- Le faible nombre de patients ayant participé au projet s’explique par le fait que le CPNPC ALK-positif métastatique est un cancer rare
- La qualité de vie a été mesurée au moyen d’un score d’utilité de l’état de santé, et les données ont été recueillies par l’entremise d’un sondage auprès des patients

Termes clés

- **ALK :** Acronyme anglais de anaplastic lymphoma kinase, c’est-à-dire kinase des lymphomes anaplasiques. Il s’agit d’un gène qui aide à la croissance cellulaire.
- **Cancer du poumon non à petites cellules (CPNPC) ALK-positif :** Type de cancer du poumon causé par des modifications (réarrangements) du gène ALK qui entraînent une croissance excessive et plus rapide des cellules cancéreuses, provoquant ainsi le cancer du poumon. Le cancer peut se propager à d’autres parties du corps (métastases), comme le cerveau.
- **Traitement ciblé :** Type de traitement anticancéreux ciblant les protéines qui contrôlent la croissance, la division et la propagation des cellules cancéreuses.
- **Inhibiteurs de tyrosine kinase (ITK) de l’ALK** : Traitements anticancéreux qui bloquent l’action d’une enzyme appelée tyrosine kinase de l’ALK et qui pourraient ralentir ou arrêter l’évolution du cancer et réduire le volume des tumeurs. Ces médicaments sont utilisés pour traiter le CPNPC ALK-positif.
  - Le crizotinib est le plus ancien ITK ciblant l’ALK (première génération)
  - L’alectinib, le brigatinib et le céritinib sont des ITK de l’ALK de deuxième génération
  - Le lorlatinib est un ITK de l’ALK plus récent (troisième génération)

Vous souhaitez en savoir plus sur ce projet?

Ce texte est un résumé en langage simple de l’article d’origine :

Rupp M, Fanton-Aita F, Snow S, Wheatley-Price P, Melosky B, Juergens RA, Chu Q, Blais N, Banerji S, Ng R, Khoudigian S, Sharma A, On PV, Liu G (2023) Lorlatinib effectiveness and quality-of-life in patients with ALK-positive NSCLC who had failed second-generation ALK inhibitors: Canadian real-world experience. Current Oncology

*Lien: **https://www.mdpi.com/1718-7729/30/7/481***

Divulgation: Cette étude a été commanditée par Pfizer Canada SRI. Le lorlatinib est approuvé pour le traitement de l’indication ayant fait l’objet de l’étude dont il est question dans le présent résumé. Ce résumé rend compte des résultats d’une seule étude. Les résultats de cette étude peuvent être différents de ceux d’autres études. Les professionnels de la santé doivent fonder leurs décisions sur l’ensemble des données probantes existantes et non pas sur les résultats d’une seule étude. La rédaction de ce résumé a été assurée par Ryan Ng, Phani Tejasvi Dantu, Shoghag Khoudigian et Arushi Sharma de IQVIA Solutions Canada Inc. et a été financée par Pfizer Canada SRI.
